# Neuroprotective, eco-friendly iron oxide nanoparticles to alleviate Parkinson’s disease symptoms and improve nursing care

**DOI:** 10.3389/fnins.2025.1692707

**Published:** 2026-01-12

**Authors:** Shuangyan Chen, Ting Jiang

**Affiliations:** Department of Neurosurgery, The Second People’s Hospital of Hunan Province (Hunan Brain Hospital), Changsha, Hunan, China

**Keywords:** Parkinson’s disease, nursing care, neuroprotective, eco-friendly, iron oxide nanoparticles

## Abstract

**Background:**

Parkinson’s disease (PD) involves motor impairment and neurochemical changes, necessitating neuroprotective agents to enhance nursing care outcomes.

**Methods:**

Iron oxide nanoparticles (IONPs) were biosynthesized via green chemistry and characterized by TEM, UV-Vis, photoluminescence, XRD, and VSM. Antioxidant activity used DPPH assay; biocompatibility involved MTT and hemolysis assays. Efficacy was tested in an MPTP-induced mouse model of PD, assessing motor function, antioxidant capacity, and dopaminergic neuron protection.

**Results:**

IONPs exhibited spherical morphology (39.0 ± 11.3 nm), zeta potential of −13 mV, and saturation magnetization of 42.32 emu/g. They showed concentration-dependent DPPH scavenging and no significant cytotoxicity or hemolysis up to 10 µg/mL. *In vitro*, IONPs improved motor performance, boosted antioxidants, and reduced apoptosis in dopamine neurons.

**Conclusion:**

Biosynthesized IONPs offer promise as a complementary PD therapy by mitigating motor deficits and neurochemical damage.

## 1 Introduction

Parkinson’s disease (PD) is one of the most prevalent neurodegenerative disorders affecting adults in middle age and beyond. Its primary clinical manifestations include the degeneration and loss of dopaminergic neurons in the substantia nigra, as well as the formation of Lewy bodies, which largely consist of misfolded *α*-synuclein (α-syn) (Khan et al., 2019). Currently, medication therapy for PD primarily relies on levodopa (L-DOPA), which effectively relieves clinical symptoms but does not slow the progression of PD. As PD advances, both the dosage and frequency of L-DOPA administration must be increased. Therefore, it is imperative to devise novel approaches to enhance the precision and effectiveness of PD treatment (Schapira, 2005; Höglinger and Trenkwalder, 2024).

The field of nanotechnology is actively advancing the development of nanoparticles (NPs) with advantageous properties for various biomedical applications. Magnetic nanoparticles (MNPs) have attracted significant interest due to their distinctive characteristics. Among them, magnetic iron oxide nanoparticles (MIONs) form a substantial class of MNPs widely used for their reactive surfaces, which can be easily altered with biocompatible coatings. MIONs are compatible with living organisms and have been widely used in diagnostics, bioimaging, and therapy (Wu et al., 2008; Laurent et al., 2010; Ajinkya et al., 2020; Vangijzegem et al., 2019). MIONs have demonstrated neuroprotective effects by reducing *α*-synuclein accumulation and inhibiting caspase-3 activation. Notably, dietary administration of MIONs reduced reactive oxygen species (ROS) levels in aged *Drosophila*, enhanced climbing performance, and extended lifespan (Imam et al., 2015). However, iron oxide nanoparticles may also induce neurodegeneration by accumulating iron in brain tissue, triggering oxidative stress, and promoting protein aggregation (Khadrawy et al., 2024). Their toxicity is influenced by various factors, including particle size, morphology, surface characteristics, concentration, and coating type (Moayeri et al., 2020).

Nanomaterials are synthesized using various physical or chemical techniques, both conventional and green. The chemicals used in these techniques have detrimental effects on the environment (Kafshgari et al., 2019; Rane et al., 2018). Consequently, the simplicity, affordability, and eco-friendly characteristics of green synthesis of nanomaterials have recently generated significant interest (Devatha and Thalla, 2018). Recent research has documented the eco-friendly production of MIONs using several biological sources, including plants, seeds, and microbes. Plant extracts are employed as non-toxic reducing and capping agents in the environmentally friendly production of nanoparticles, known as green synthesis, replacing hazardous reducing chemicals. The antidepressant action of iron oxide nanoparticles produced using curcumin as a reducing agent was observed (Bolade et al., 2020; Priya et al., 2021).

*Mentha piperita*, commonly known as peppermint, is a highly aromatic herb belonging to the Lamiaceae family. It is a perennial plant with smooth, hairless leaves. This specific species has been cultivated in temperate and subtropical locations for more than a thousand years. Mints are highly prized for their commercial value and unique fragrance (Almatroodi et al., 2021; Silva, 2020). *M. piperita* is a highly nourishing green vegetable abundant in essential vitamins and minerals. Flavonoids, phenols, and terpenoids are primarily located in the leaves of the plant, although diterpenes can be found in both the leaves and stems (Mainasara et al., 2018).

The current study presents the green Mentha piperita–mediated synthesis of MIONs, which can serve as a neuroprotective platform for Parkinson’s disease and have potential relevance for patient care and outcomes for nurses. For the first time, these MIONs synthesized via plant extraction are translated through an inclusive pipeline comprising extensive physicochemical and magnetic characterization and thorough *in vitro* biocompatibility and antioxidant assessments and culminate in a targeted preclinical evaluation in the MPTP animal model. This synthesis-to-preclinical study establishes a nanomaterial production process in an environmentally sustainable manner and a translational neurointervention-to-preclinical approach, providing an environmentally sustainable route for nanomaterial production and establishing a translational pathway toward future human-oriented and clinical investigations.

## 2 Experimental

### 2.1 Chemicals and reagents

FeCl_2_· 4H_2_O, 70% ethanol, and DMSO were purchased from Merck (Zurich, Switzerland). DMEM, fetal bovine serum (FBS), distilled water, and Pen/Strep were obtained from Gibco by Life Technologies (USA).

### 2.2 Instrumentation

TEM imaging was performed using a JEM-1230 electron microscope (JEOL, Tokyo, Japan). DLS and zeta potential measurements were carried out using a Zetasizer Nano Series (Nano ZS, Malvern Instruments, UK). The XRD pattern was recorded with a PANalytical X’Pert PRO X-ray diffractometer (PANalytical, The Netherlands). FTIR spectroscopy was conducted using an FTIR spectrometer (Edwards High Vacuum, Crawley, Sussex, England). The absorbance of the biological solutions was then measured using a microplate reader (Tecan, Switzerland).

### 2.3 Biosynthesis of IONPs

The fresh leaves of the *M. piperita* plant were collected, weighed, dehydrated, ground into a fine powder, and then extracted with an aqueous solution in an Erlenmeyer flask. MIONPs were synthesized based on previous reports with slight modifications (Yusefi et al., 2020). A 1 mM solution of FeCl_2_.4H_2_O was prepared by dissolving 20 mg of the salt in 100 mL of distilled water, and the resulting solution was mixed with the obtained extract (1 volume of the salt solution to 2 volumes of the extract). The reaction mixture was heated for 20 s using a microwave. The reaction mixture was centrifuged at 5,000 rpm for 30 min, and the resulting pellet was separated, washed, and stored for analysis.

### 2.4 Physical characterizations

DLS provides data on particle size and the degree of size variation, known as the polydispersity index (PDI). The Zetasizer Nano Series was used to measure the hydrodynamic size and zeta potential of IONPs at 25 °C. TEM provides detailed images of each particle, providing valuable insights into the size and morphology of biosynthesized NPs. The dimensions and structure of IONPs were assessed using TEM. In summary, a small amount of IONP solution (14 mg/mL) was applied to the grid, and any excess liquid was removed using filter paper. Before examining the samples, the grid was left to dry at ambient temperature. XRD was employed to identify the phases and ascertain the crystalline structures of nanoparticles. The IONPs were analyzed using an X-ray diffractometer with a Cu Kα radiation source (*λ* = 1.54 Å) and a Cu anode. The applied accelerating voltage and current were 45 kV and 30 mA, respectively. The scanning was performed at 25 °C over the angular range 20° ≤ 2θ ≤ 79.99°. The surface functional groups of the IONPs were evaluated using FTIR spectroscopy. The FTIR scan was conducted.

### 2.5 *In vitro* studies

#### 2.5.1 Antioxidant activity

The antioxidant potential of the biosynthesized IONPs was evaluated using the DPPH assay protocol, with slight modifications from a previous study (Kunjan et al., 2024). Various concentrations (5, 10, 15, 20, 25, 30, 40, and 45 μg/mL) of IONPs in 2 mL of solution were incubated with 2 mL of DPPH solution (0.1 mM) for 20 min at 37 °C. Finally, the absorbance of the mixture was read at 517 nm.

#### 2.5.2 Hemocompatibility

The hemocompatibility of the biosynthesized MIONPs was evaluated based on their effects on RBCs and the induction of hemolysis. Various amounts (1, 5, 10, 15, 20, 30, and 40 μg/mL) of IONPs were incubated with a diluted RBC mixture for 1 h at 37 °C. The mixture was then centrifuged at 3,000 rpm for 5 min, and the absorbance of the supernatant was measured at 545 nm using a microplate reader.

#### 2.5.3 Cytocompatibility

The cytocompatibility of the biosynthesized IONPs was evaluated using the MTT assay, a colorimetric method that detects metabolically active cells. Cells (10 × 10^3^ cells) were suspended in 100 μL of DMEM/FBS/Pen/Strep, seeded into 96-well plates, and incubated at 37 °C for 24 h. Then, the cells were exposed to different concentrations of IONPs (1, 5, 10, 15, 20, 30, and 40 μg/mL) and incubated again at 37 °C for 24 h. Next, MTT solution (0.5 mg/mL) was added to each well and incubated for 4 h. Finally, DMSO was added, and absorbance was measured at 570 nm using a microplate reader.

### 2.6 Preclinical evaluation

#### 2.6.1 Model induction

The animal studies were conducted on 18 C57BL/6 J female mice (12 weeks old, 21.5 ± 0.4 g) in accordance with approved guidelines. The mice were housed under standard, controlled conditions with a 12/12 light/dark cycle and a constant temperature of 25 ± 2 °C. They had access to standard food pellets and tap water *ad libitum* throughout the experiment. The animals were randomly assigned to three groups (*n* = 6 per group): a healthy group (no PD induction, treated with PBS), a negative control group (PD induced, treated with PBS), and a nanoformulation group (PD induced, treated with biosynthesized MIONPs). Experimental Parkinsonism was induced via intraperitoneal injections of MPTP (15 mg/kg in 0.9% saline), administered four times at 3-h intervals.

#### 2.6.2 Behavioral studies

##### 2.6.2.1 Pole climb-down test

Motor coordination and bradykinesia in the animals under treatment were evaluated using the pole-climb-down test. It involved using a vertical wooden rod with a rough surface, measuring 50 cm in height and 1 cm in diameter, which was securely attached to a platform. The mouse was positioned at the top of the pole with its head facing upward. The time it took for the mouse to turn around and descend to the base was recorded on video. If a mouse was unable to successfully climb down or slipped, a default value of 120 s was assigned.

##### 2.6.2.2 Stride length

Stride length, or gait analysis, was employed to assess typical gait functions and walking synchronization. The apparatus consisted of a straight hallway measuring 2.5 feet in length and 8.5 cm in width, with boundary walls that were 8 cm high. The floor was covered with white paper. The hind limbs were coated with a water-soluble, non-toxic ink that allowed the animal to move freely. The footprints were allowed to dry naturally, and the distance between the inner toes of two successive hind limbs on the same side was measured using a Vernier caliper.

#### 2.6.3 Neurochemical measurements

The mice were administered anesthesia using a mixture of ketamine (100 mg/kg) and xylazine (10 mg/kg). Subsequently, the mice were humanely terminated by cervical dislocation. Each mouse was beheaded, and its brain was obtained using sterile surgical instruments in a clean area beneath a dissecting microscope. The mouse brain was promptly rinsed with ice-cold phosphate-buffered saline. The cerebral hemisphere was cryogenically preserved using liquid nitrogen for antioxidant analysis. Dopamine levels in the midbrain of mice were assessed by high-performance liquid chromatography (HPLC), with three per group. The midbrain was pulverized in phosphate buffer and then centrifuged (10,000 g at 4 °C) to collect the supernatant above the sediment. A standard concentration of dopamine (Merck, H8502), 0.1 mg/mL, was used. The mobile phase consisted of a mixture containing 0.01% EDTA (adjusted to pH 4.0 using 100% acetic acid), 2% methanol (volume/volume), and 7% acetonitrile (volume/volume). Before use, all HPLC solutions were filtered twice through 0.2 μm membranes and degassed, as described in a prior study (Karaagac et al., 2010). The mobile phase flowed at 0.8 mL/min, and the UV detector was set to 340 nm. The final concentration of dopamine was expressed as micrograms per milligram of brain tissue.

#### 2.6.4 Molecular studies

The brain was washed in sterilized, ice-cold PBS after procurement. The brain tissue was homogenized in TRIzol reagent by repeatedly pipetting the tissue manually with micropipette tips of decreasing diameter. Total RNA was extracted using the isopropanol-chloroform method. Then, 1 μg of RNA with a 260/280 ratio of 1.8–2 was used for complementary DNA (cDNA) synthesis. The cDNA synthesis was performed using the commercially available RevertAid cDNA synthesis kit. The cDNA was diluted 4 × in RNase/DNase-free water and stored at −20 °C. Real-time qRT-PCR was performed using CFX 96 plates with a SYBR Green probe. The mRNA expression was determined by calculating the fold change relative to the control using the 2 − ΔΔCt formula, where ΔCt represents the difference in cycle threshold (Ct) values between the target and reference genes. The fold change was calculated using Ct values generated by the software.

### 2.7 Statistical analysis

The results were reported as the mean value ± the standard error of the mean (SEM). The data were analyzed using one-way analysis of variance (ANOVA), followed by the Duncan *post hoc* test to determine group differences. The significance level was set at a *p*-value of 0.05.

## 3 Results and discussion

### 3.1 Eco-friendly IONP characteristics

**Figure 1:**
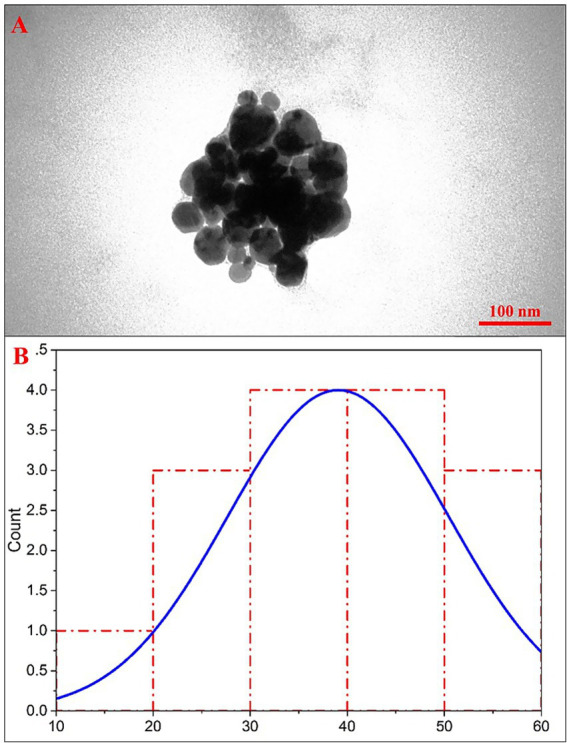
**(A)** TEM image and **(B)** size distribution histogram of the eco-friendly IONPs.

TEM imaging was used to visualize the eco-friendly IONPs, evaluate their morphology, and measure nanoparticle diameter and size distribution. The results (Figures 1A,B) show that the eco-friendly IONPs have a spherical morphology with a size of 39.0 ± 11.3 nm. The morphology and size of nanoparticles are affected by various parameters, including the source of the starting materials, incubation time, temperature, and surfactants. The field of green synthesis of iron nanoparticles is a captivating area of research that has become significant for its reliable, sustainable, and environmentally friendly approach to nanoparticle synthesis. This is further enhanced by the convenient accessibility of plant materials and their medicinal importance.

Biological materials, such as microbes and plants, are considered more cost-effective and eco-friendly alternatives to physical and chemical synthesis. Biological extracts have been utilized to generate iron nanoparticles of various shapes and sizes. Microorganisms (such as bacteria, fungi, and algae) and plant extracts have been used to produce IONPs via green synthesis. This is possible because these microorganisms and plants contain a variety of metabolites and biomolecules. Biologically generated IONPs possess superior physical and biochemical properties compared to those synthesized using physical or chemical methods. IONPs possess magnetic properties, as well as thermal and electrical conductivity.

Iron nanoparticles, with a typical size of 10–20 nm, can exhibit a distinct form of magnetism known as superparamagnetism. These substances are not harmful and can be easily dispersed to reach specific areas, making them ideal for effectively delivering drugs to their intended targets. Researchers have investigated the use of IONPs generated by green methods for a wide range of biotechnological applications. The IONPs demonstrated both antibacterial and anticancer activities. IONPs negatively affect cell viability, division, and metabolic activity. Iron nanoparticles have been used to purify and immobilize a wide range of enzymes and proteins. IONPs have demonstrated promise for the bioremediation of a wide range of organic and inorganic contaminants (Kumar et al., 2023; Zúñiga-Miranda et al., 2023; Nadeem et al., 2022).

Kiwumulo et al. (2022) used *Moringa oleifera* to biosynthesize FeNPs and reported that the NPs possessed a granular, homogenous, spherical morphology with an average diameter of approximately 16 nm. They proposed that these FeNPs could be used in drug delivery. Mohamed et al. (2023) synthesized green iron oxide nanoparticles using clove and green coffee (g-coffee) extracts as absorbents for Cd^2+^ and Ni^2+^ ions. They reported that clove-mediated FeNPs displayed a rod-shaped and agglomerated morphology, while g-coffee-mediated FeNPs exhibited a pyramidal-shaped morphology and were clearly larger in size.

**Figure 2:**
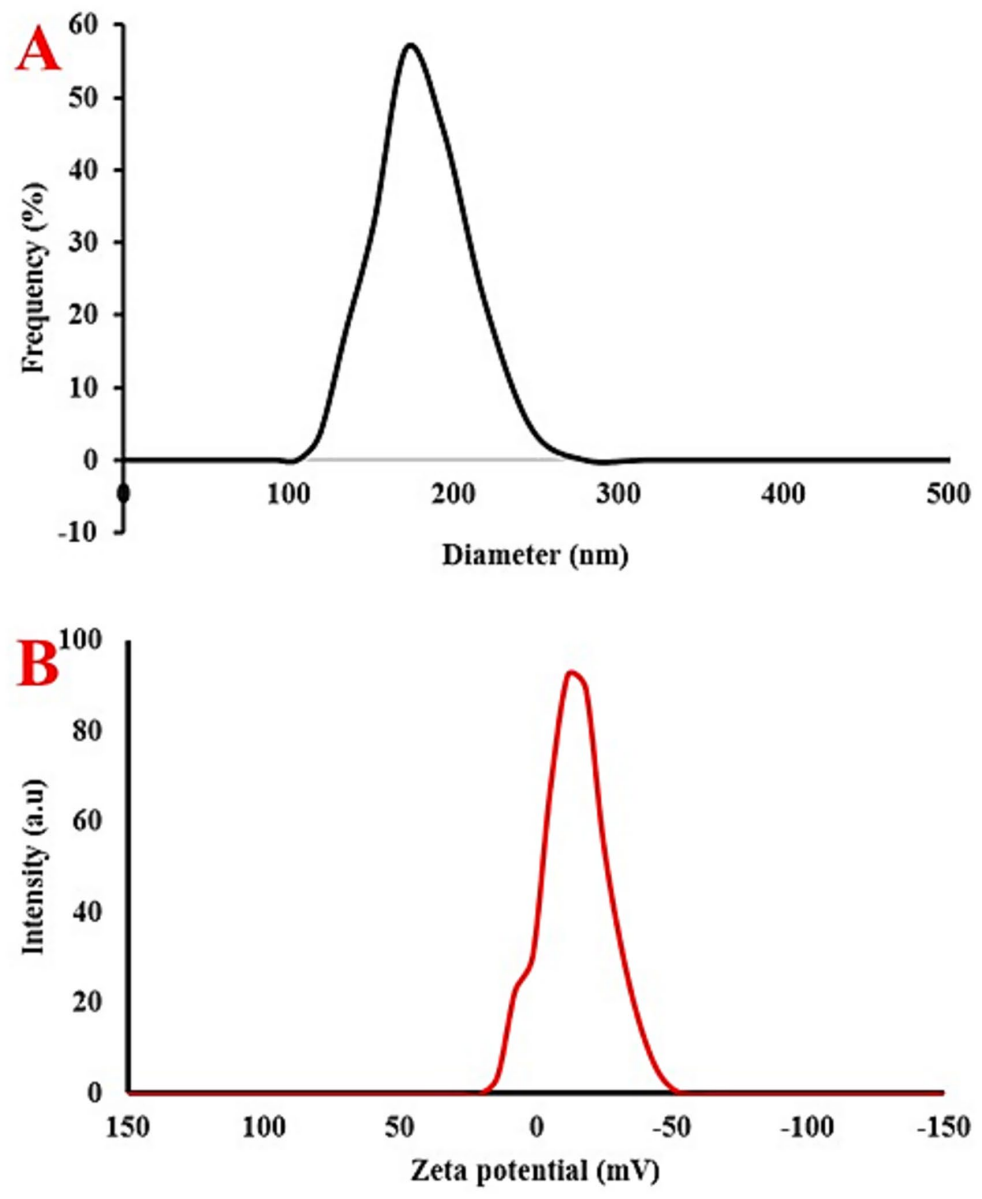
**(A)** Hydrodynamic diameter and **(B)** zeta potential of eco-friendly IONPs.

The zeta potential reflects the potential difference between the electric double layer (EDL) of electrophoretically mobile particles and the dispersant layer surrounding them at the slipping plane. The phrase “electrokinetic potential” refers to the potential at the slipping or shear plane of a colloid particle that is in motion due to an electric field (Vold, 1982; Delgado et al., 2005). Thus, the particle size distribution and the magnitude of the electric charge on the particle surface are determined. Additionally, a zeta sizer was used to determine particle size. The intensity was used to scan the size distribution. Nevertheless, due to variations in the dispersion coefficient and cluster formation, it failed to yield precise results. The hydrodynamic diameter and zeta potential of the eco-friendly IONPs were measured using the DLS technique. The results showed that the hydrodynamic diameter of the eco-friendly IONPs was around 171 nm (Figure 2A). Lakshminarayanan et al. applied *Bauhinia tomentosa* leaf extract to synthesize FeNPs and reported that the NPs exhibited −16 mV. In the present study, we observed a zeta potential of −13 mV, indicating successful nanoparticle formation (Figure 2B).

**Figure 3:**
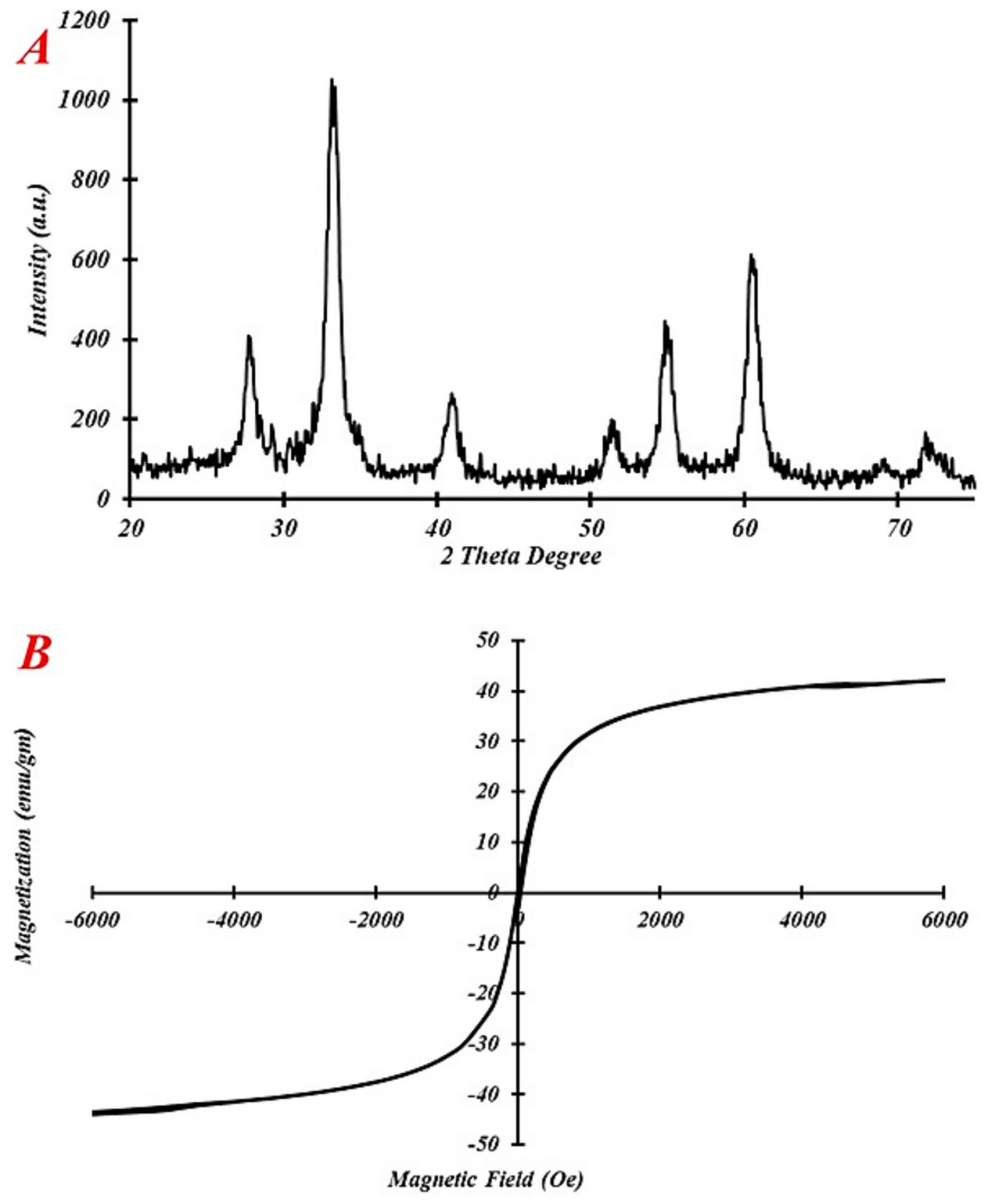
Optical properties of eco-friendly IONPs: **(A)** UV–Vis spectrum and **(B)** photoluminescence spectrum.

The use of UV–Vis spectrometry has allowed the identification of specific NPs produced during a color change, as indicated by the absorption spectra. An optical spectroscopy technique was employed to measure the wavelength range from 200 to 400 nm. The results (Figure 3A) show a prominent peak at 244 nm, indicating the presence of NPs. Figure 3B shows the photoluminescence spectrum of the synthesized eco-friendly IONPs, with a strong luminescence band located at 435 nm. The observed photoluminescence peak can be attributed to the electronic transition within the iron oxide (*α*-Fe_2_O_3_) (Lassoued et al., 2018). Previously, it was shown that the distinctive surface plasmon resonance band of Fe_3_O_4_ primarily appears at wavelengths between 190 and 250 nm. This was observed when varying the quantities of metal ions and the volumes of plant extracts. The presence of a distinct signal at 244 nm provides evidence for the existence of the FeNPs (Rufus et al., 2016).

**Figure 4:**
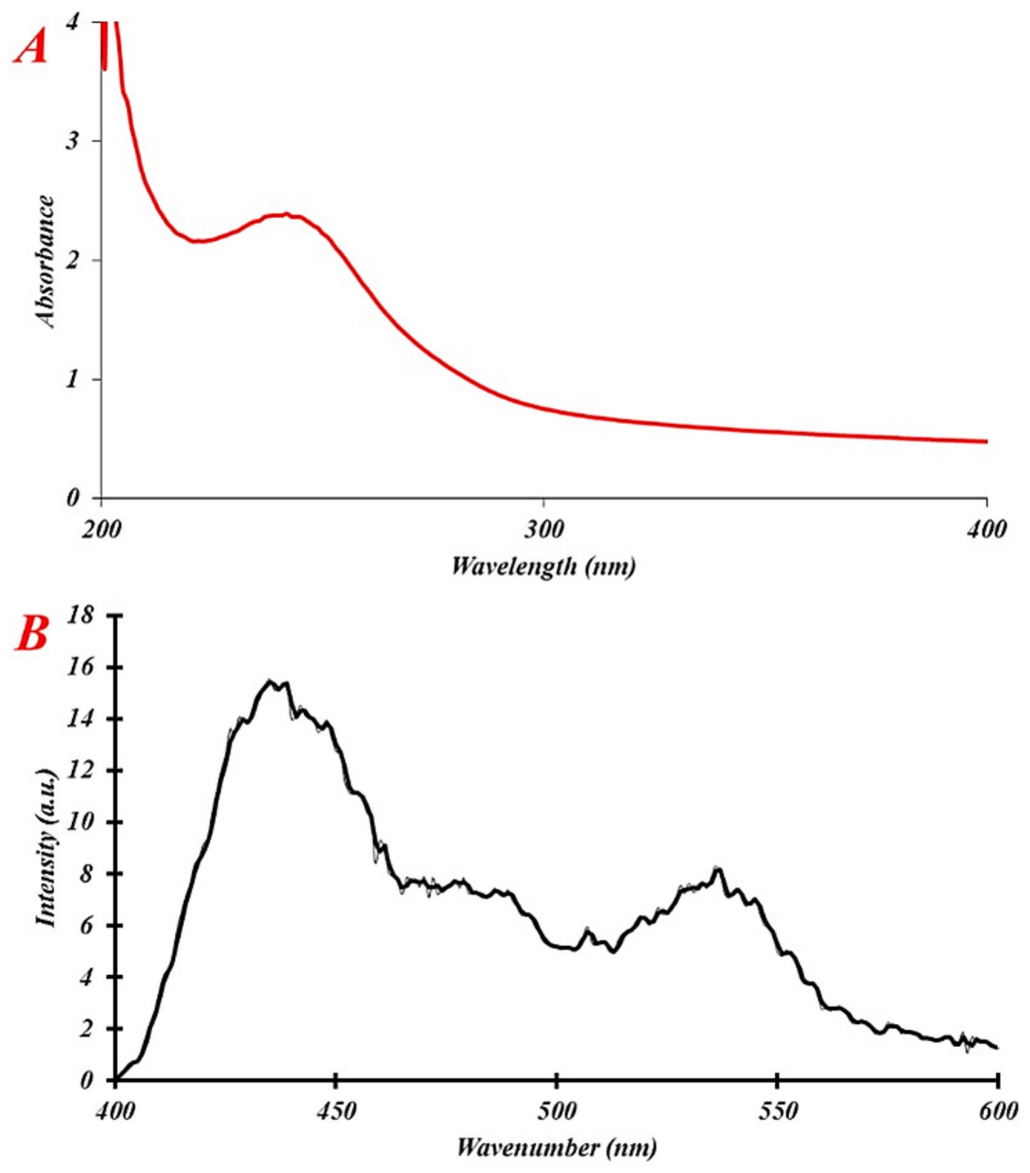
**(A)** XRD pattern and **(B)** vibrating sample magnetometer of eco-friendly IONPs, wavenumber (cm^−1^).

The crystallinity of the eco-friendly IONPs was evaluated using XRD, as shown in Figure 4A. The peaks at approximately 27.7°, 33.1°, 41.0°, 51.3°, 54.8°, and 60.4° correspond to the 012, 104, 110, 113, 024, 116, and 018 crystalline structures. The obtained XRD pattern refers to the Joint Committee on Powder Diffraction Standards (JCPDS) File No. 87–1,166 (Murugan et al., 2018).

The VSM was used to examine the magnetic characteristics of eco-friendly IONPs. Figure 4B depicts the magnetic properties of the eco-friendly IONPs at room temperature. The magnetization curves clearly indicate a high saturation magnetization (Ms) value of 42.32 for eco-friendly IONPs. Based on the magnetization curves, eco-friendly IONPs exhibit a high Ms., which can be attributed to a greater number of IONPs captured within the extract. Magnetic hysteresis refers to the lag, or delay, in the magnetization of a material when it is magnetized and then demagnetized (Izadiyan et al., 2020). Karaagac and Kockar used orthogonal optimization of co-precipitation parameters, determining an optimized combination of parameters (including an [Fe^2+^]/[Fe^3+^] ratio, iron concentration, base concentration, and reaction time) that provided maximum room-temperature saturation magnetization (Ms = 69.83 emu·g^−1^) for air-synthesized superparamagnetic iron oxide nanoparticles (Karaagac and Kockar, 2016). This suggests that appropriately optimizing co-precipitation parameters can yield significantly enhanced Ms. and provides a reference for our IONP study.

Studies on hydrothermal growth show that reaction parameters, particularly temperature, reaction time, and precursor concentration, consistently regulate particle size distribution and crystallinity, and that the optimal selection of these parameters can increase saturation magnetization; therefore, a systematic parameter study (temperature/time/concentration) should be able to improve Ms. for this green synthesis (Ozel et al., 2015).

In line with previous reports, our green co-precipitation synthesis produced IONPs whose magnetic and structural features varied with synthesis conditions. As observed in other air-co-precipitated systems, modest changes in precursor concentration and the Fe^2+^/Fe^3+^ ratio can alter crystallinity and particle size, which, in turn, affect the measured Ms. (Karaagac et al., 2010). In another study, the results showed that synthesis temperature was strongly correlated with IONP crystallinity and size, with higher synthesis temperatures generally producing more ordered crystals and XRD-defined particle size and, in most cases, higher overall measured saturation magnetization (Karaagac et al., 2011).

In another study, research on iron-oleate precursors showed that the amount of oleic acid in the precursor affects the thermal decomposition behavior and surface coating, producing systematic changes in crystal size and saturation magnetization; lower oleic acid amounts tend to produce larger crystallites and altered $M_{s}$ (Ozel et al., 2013).

**Figure 5:**
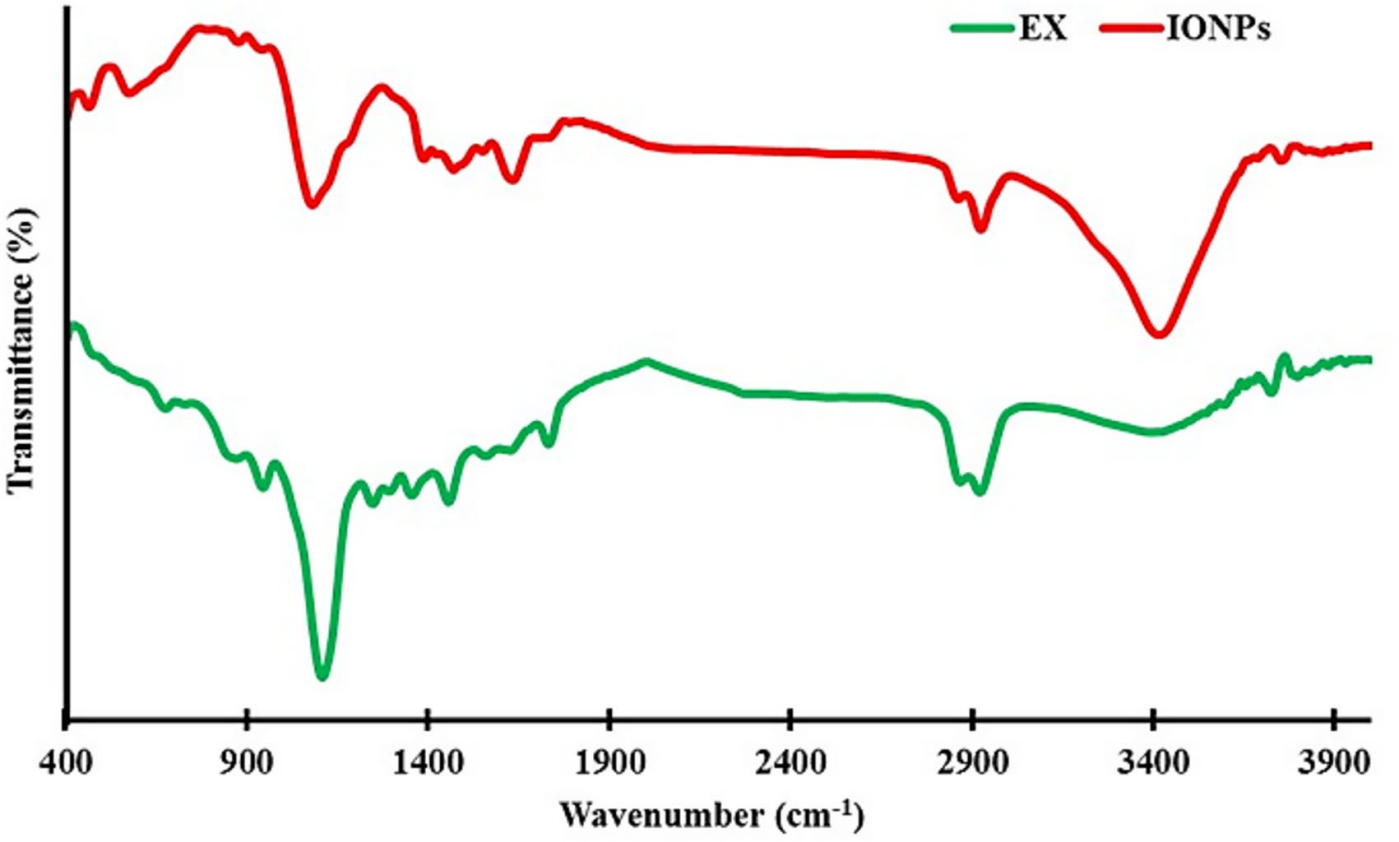
FTIR spectrum of pure extract and eco-friendly IONPs. 2 figure.

The surface functional groups and the presence of the extract’s active components on the synthesized eco-friendly IONPs were evaluated using FTIR spectroscopy. The results (Figure 5) showed that the synthesized eco-friendly IONPs exhibited characteristic peaks of the extract. The peak around 3,426 cm^−1^ corresponds to OH stretching, 2,924 cm^−1^ corresponds to CH_3_ and CH_2_ functional groups, and the peak around 1,635 cm^−1^ can be related to the C=C aromatic ring of the phytochemicals. These observations revealed that the phytochemicals in the extract are adsorbed onto the IONPs during green synthesis and serve as capping agents, modifying the IONP surface.

### 3.2 Biological characteristics of eco-friendly IONPs

#### 3.2.1 *In vitro* studies

**Figure 6:**
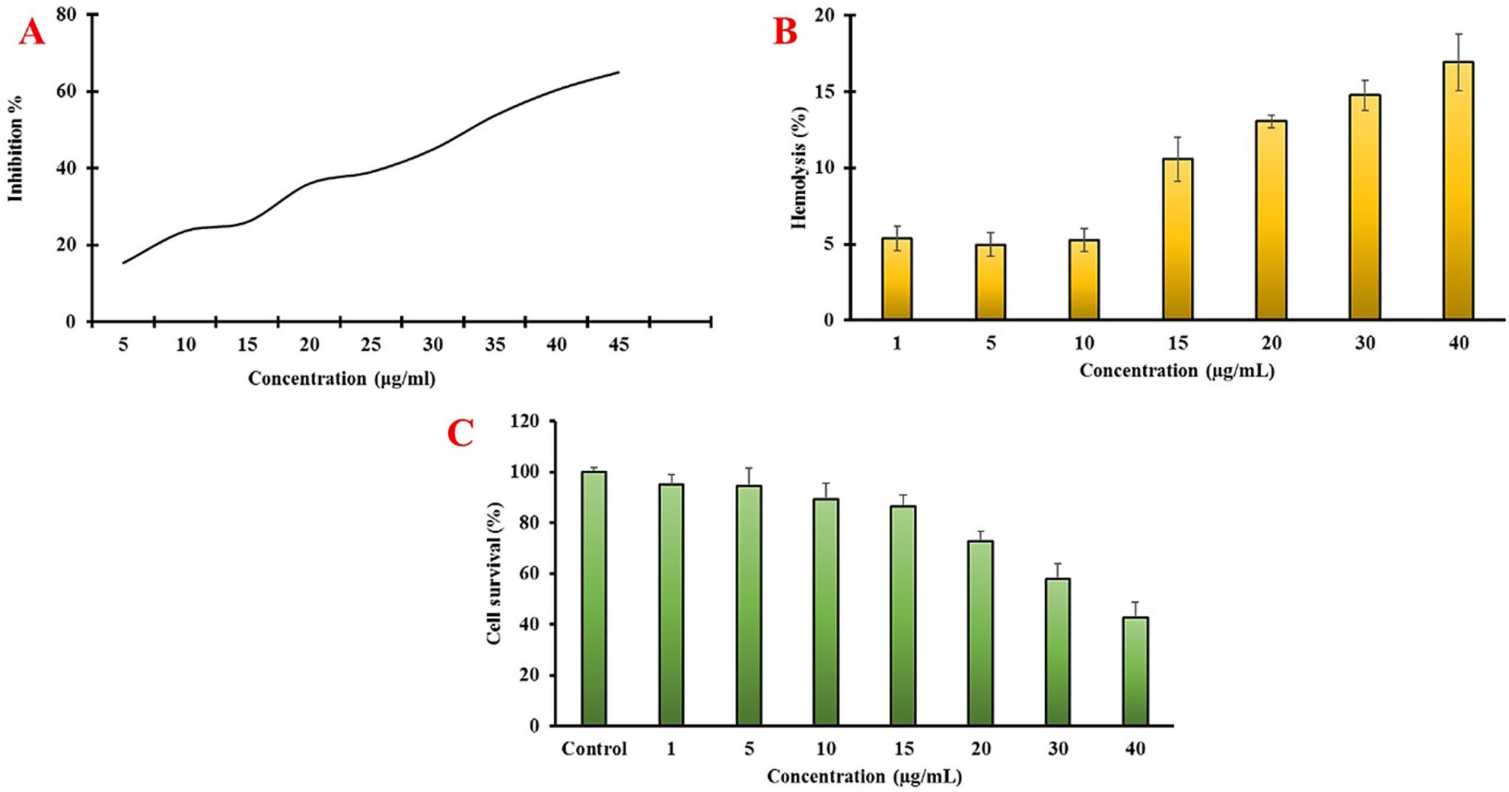
*In vitro* biological activities of eco-friendly IONPs: **(A)** antioxidant activity against DPPH free radicals, **(B)** hemolysis of RBCs, and **(C)** cytocompatibility results.

During PD development, the substantia nigra is damaged by ROS, which induce lipid peroxidation, protein oxidation, and DNA oxidation. This condition appears to be primarily caused by alterations in brain iron levels, mitochondrial dysfunction, activation of monoamine oxidase (MAO), or changes in the antioxidant defense system. In addition to using purified antioxidant molecules, numerous studies have explored the potential of unrefined plant extracts to address symptoms resembling those of Parkinson’s disease (PD) and the resulting structural and biochemical changes observed in PD models (Chang and Chen, 2020; Percário et al., 2020). This is primarily due to the combined antioxidant properties of these extracts (Percário et al., 2020). The antioxidant potential of eco-friendly IONPs was evaluated using the DPPH assay method. The results (Figure 6A) showed that the NPs exhibited concentration-dependent radical-scavenging activity.

The hemocompatibility of eco-friendly IONPs was evaluated using the RBC hemolysis assay. The results (Figure 6B) show that eco-friendly IONPs did not induce significant hemolysis at concentrations up to 15 μg/mL. Hemolysis at 1, 5, and 10 μg/mL was below 8%, indicating they are hemocompatible. The cytocompatibility of the eco-friendly IONPs was evaluated using the MTT assay, and the results are presented in Figure 6C. The results revealed that the eco-friendly IONPs were not cytotoxic at concentrations up to 10 μg/mL, whereas higher concentrations were cytotoxic.

#### 3.2.2 Animal study results

**Figure 7:**
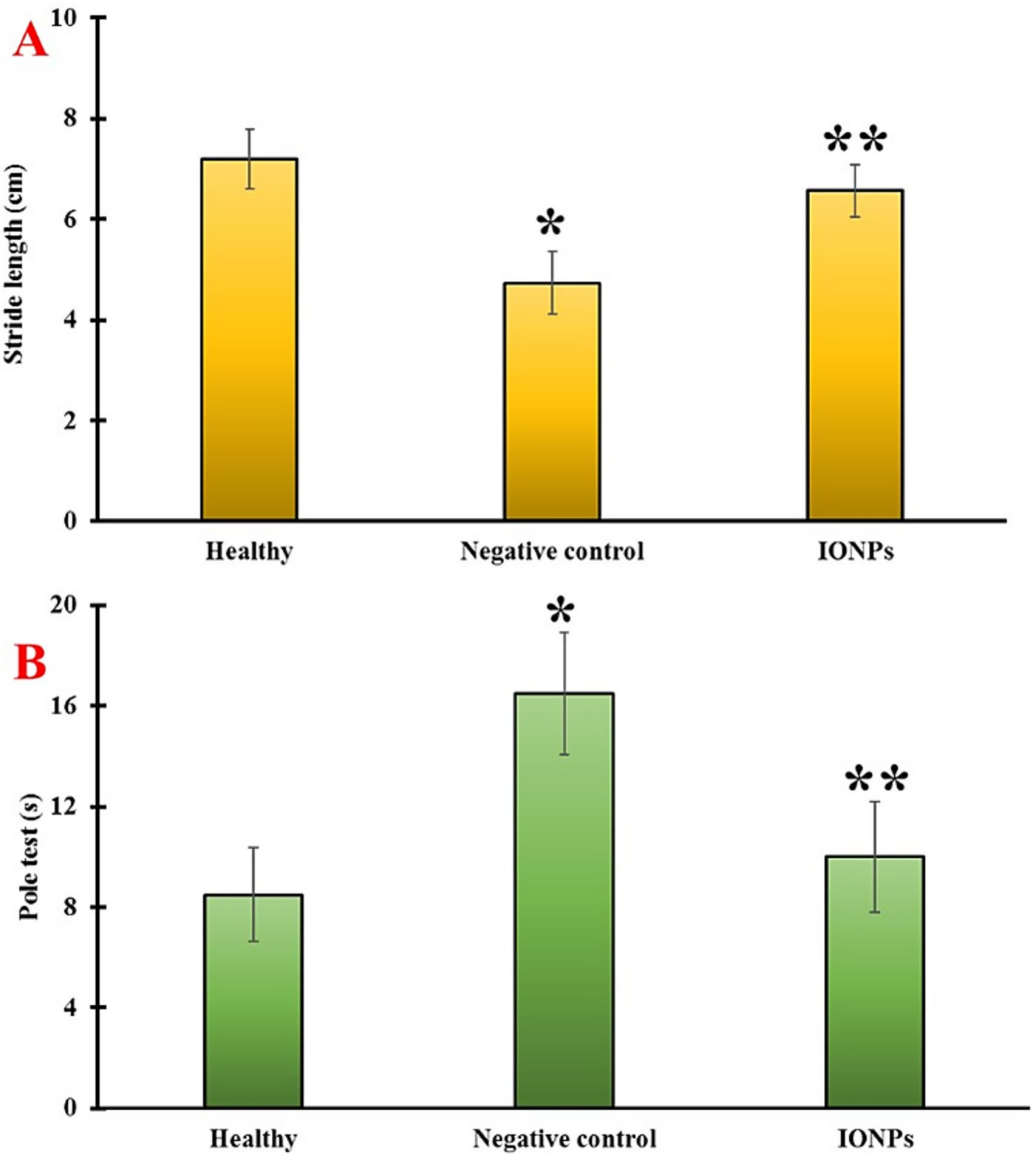
Behavioral test results. **(A)** Stride length and **(B)** pole test. *p < 0.05 in comparison with healthy animals. ***p < 0.05 in comparison with the negative control.

Motor dysfunction under PD induction was assessed using the pole and stride tests. The results (Figure 7A) showed that MPTP significantly reduced stride length compared with healthy animals (*p* < 0.05), whereas treatment with eco-friendly IONPs recovered motor dysfunction induced by PD. The results (Figure 7B) indicated that MTPT increased the time spent on the pole during the pole test (*p* < 0.05). On the other hand, treatment with eco-friendly IONPs recovered impaired motor function and reduced the pole test score (*p* < 0.05 compared with the negative control group). The functional impairments observed in mice inflicted with MPTP were consistent with prior research findings (Sedelis et al., 2001; Luchtman et al., 2009; Kurosaki et al., 2004). Recent behavioral evaluations have demonstrated that MP treatment for 21 days enhances locomotor activity in MPTP-challenged mice. The use of eco-friendly IONPs improved motor deficits, as evidenced by decreased climb-down time and increased locomotor activity, as assessed using pole, stepping, and stride length tests. The results of this investigation align with a prior report indicating the therapeutic potential of an herbal extract in reducing the severity of motor dysfunction (Kim et al., 2016; da Costa et al., 2017; Chang et al., 2019).

A single moderate dose of environmentally friendly IONPs was selected for *in vivo* administration in this study based on *in vitro* cytocompatibility and hemocompatibility assessments, which documented no significant toxicity up to 10 μg/mL. Comparable dose concentrations have previously been shown to be safe and neuroprotective in both healthy and diseased rodent models using either green-synthesized or curcumin-capped IONPs (Khadrawy et al., 2024; Moayeri et al., 2020). The dose selected in the current work was specifically chosen to be well below the known maximum tolerated dose (MTD) of IONPs in mice, which can vary significantly in value between 1 and 50 mg/kg and is highly dependent on nanoparticle size, coating, and route of administration (Laurent et al., 2010; Ajinkya et al., 2020).

The selected dose was administered once daily for 21 days to establish a steady state, allowing repeated exposures while still ensuring safety. A complete assessment of the dose–response, which would ideally be performed in all studies, was beyond the limitations of this study due to both resource and ethical considerations. However, the behavioral, biochemical, and molecular improvements observed throughout this study indicate that the selected dose was effective and well-tolerated. Future studies will focus on systematic dose optimization and long-term safety investigations to better define the therapeutic window of these environmentally friendly IONPs.

In addition, prior research utilized only open field and rotarod tests to assess motor capabilities in animals (Xu et al., 2023). However, in the present study, supplementary behavioral tests were employed to accurately gage the extent of the disease.

**Figure 8:**
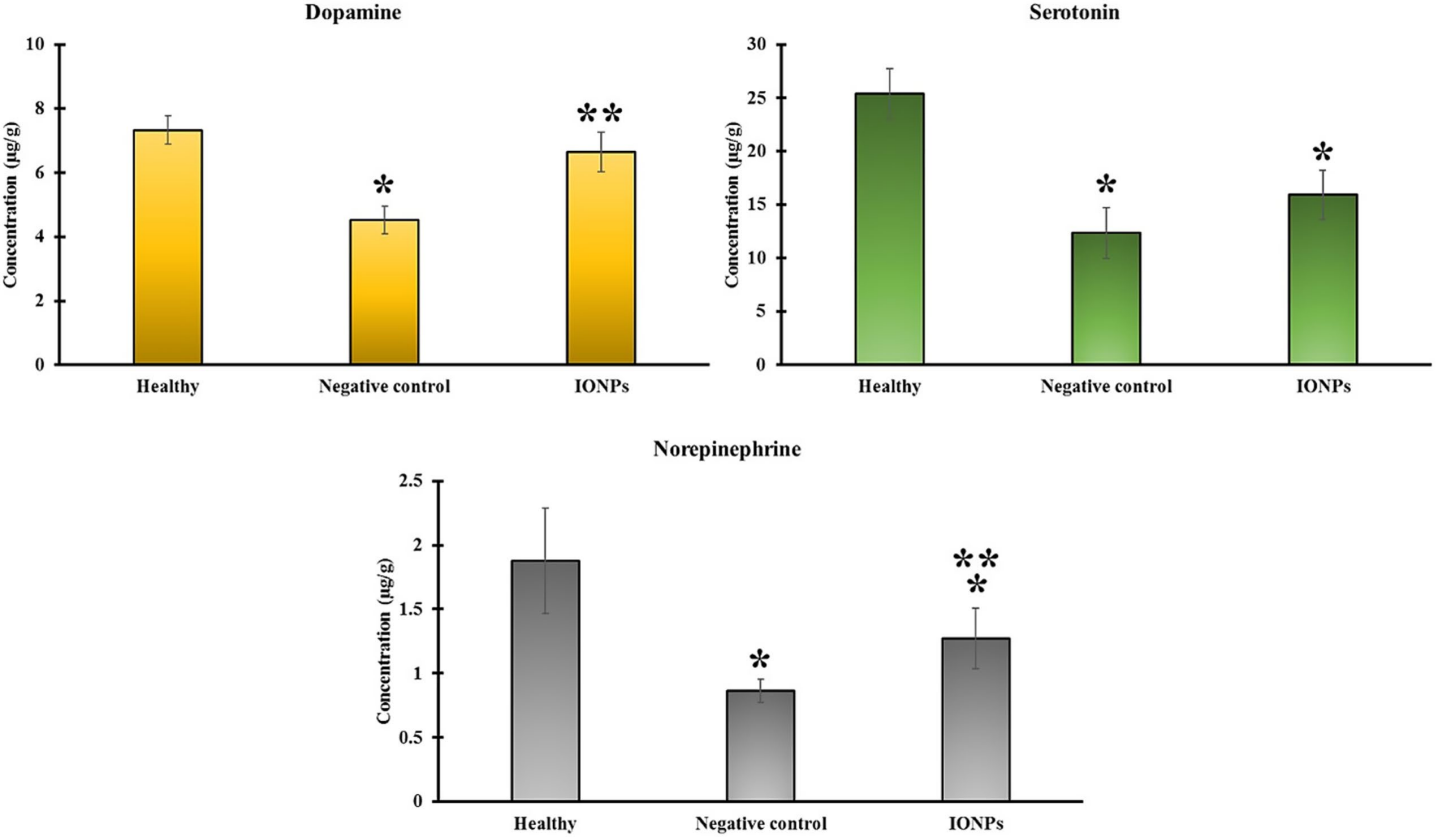
Effect of eco-friendly IONPs on levels of serotonin, norepinephrine, and dopamine in the midbrain of the MPTP-induced PD animal model. *p < 0.05 compared to healthy animals. ***p < 0.05 in comparison with the negative control.

The biochemical analysis (Figure 8) shows that the MTPT neurotoxin disrupted neurotransmitter levels (dopamine, serotonin, and norepinephrine) in the midbrain of animals, which were significantly reduced compared to the negative control (*p* < 0.05). MTPT reduced dopamine levels from 7.33 ± 0.44 μg/g in healthy animals to 4.52 ± 0.42 μg/g, whereas eco-friendly IONPs restored them to 6.64 ± 0.62 μg/g. MTPT reduced the serotonin level from 25.35 ± 2.36 μg/g in healthy animals to 12.34 ± 2.35 μg/g, while eco-friendly IONPs recovered the dopamine level to 15.90 ± 2.30 μg/g.

Norepinephrine, commonly known as noradrenaline, functions as both a neurotransmitter and a hormone. As a neurotransmitter, it acts as a chemical messenger that facilitates the transmission of nerve signals from one nerve cell to another, as well as from nerve cells to muscle or gland cells. Cortisol is a hormone secreted by the adrenal glands, which are glandular structures resembling hats located on the upper poles of each kidney. Norepinephrine is synthesized from dopamine and functions as a neurotransmitter. It is synthesized by neurons in the brainstem and in a region adjacent to the spinal cord. MTPT reduced dopamine levels from 1.87 ± 0.41 μg/g in healthy animals to 0.86 ± 0.09 μg/g, whereas eco-friendly IONPs restored them to 1.27 ± 0.23 μg/g.

**Figure 9:**
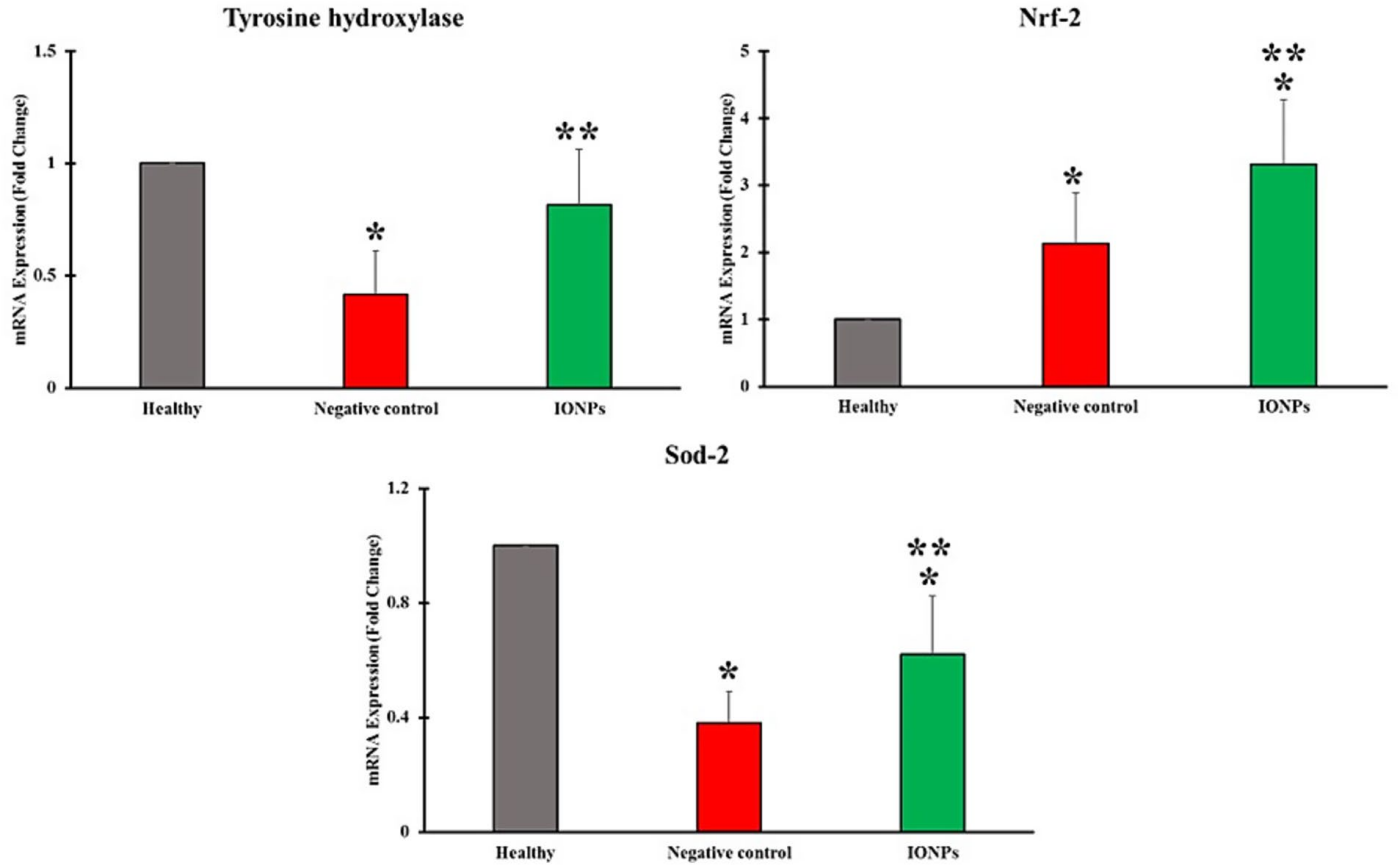
RT-qPCR results for tyrosine hydroxylase, Nrf-2, and Sod-2. Data are presented as mean ± SEM; *n* = 6 sections per group, *p < 0.05 vs. healthy, **p < 0.01 vs. control. One-way ANOVA followed by Bonferroni *post-hoc* analysis.

Using RT-qPCR, the genes tyrosine hydroxylase, Nrf2 (nuclear factor E2-related factor 2), and superoxide dismutase (SOD) were evaluated. The results (Figure 9) showed that MPTP-induced PD significantly reduced TH expression (*p* < 0.05). TH plays a critical role in DA biometabolism, converting L-tyrosine to L-DOPA, which can subsequently be converted to DA.

Our findings revealed that treatment with eco-friendly IONPs recovered the damage induced by MTPT and elevated TH expression. Nrf2 and SOD have central roles in antioxidant defense against oxidants and inflammation. The results indicated that treatment with eco-friendly IONPs enhanced antioxidant capacity and protected cells against oxidative stress. The observed findings are consistent with previous studies. Khadrawy et al. (2024) applied the green chemistry method to synthesize curcumin-capped IONPs and used them as neuroprotective agents in a rat model of Parkinson’s disease. They reported that the synthesized IONPs could prevent motor deficits by restoring dopamine and norepinephrine levels. They proposed that the observed effects are attributable to the antioxidant and radical-scavenging properties of the synthesized IONPs. In another study, Anjum et al. used Mentha piperita extract in a mouse model of Parkinson’s disease to evaluate its neuroprotective potential. They also observed potent neuroprotective activity, which may be due to the strong antioxidant activity.

Despite the preclinical nature of this study, the preservation of motor performance and dopaminergic indices in treated subjects after eco-friendly IONP treatment suggests potentially downstream implications for patient management in nursing practice. In patients with PD, measurable improvements in gait, bradykinesia, and overall motor performance can decrease fall risk and increase independence in activities of daily living (ADLs) while also reducing the frequency and intensity of hands-on care and nursing staff observation. At the same time, therapies with stabilizing effects on motor symptoms will also assist in safely titrating medications and reducing acute altered symptoms. Most importantly, these real and useful indicators of clinical care cannot be interpreted from rodent endpoints. To definitively assess the nursing-relevant benefits, future prospective translational studies would require functional independence endpoints, fall impact, and caregiver strain, alongside safety/biodistribution monitoring (serum iron metrics or liver/spleen histopathology) and long-term follow-up.

This study has several limitations that should be acknowledged. Direct quantification of regional brain iron was not performed, which could limit the assessment of nanoparticle retention through localized iron accumulation. In addition, there was no pharmacological positive control, so the effect size cannot be benchmarked against standard therapies. Furthermore, this study did not assess IONP biodistribution, and these limitations can be addressed in future studies.

## 4 Conclusion

In the present study, we aimed to synthesize potent, multifunctional nanoformulations using an eco-friendly approach. We used Mentha piperita extract to synthesize eco-friendly IONPs. The resultant eco-friendly IONPs exhibited promising physicochemical and biological activities. We observed that the eco-friendly IONPs prevented neurotoxicity and PD induced by MTPT. The eco-friendly IONPs ameliorated motor impairment by increasing antioxidant capacity and protecting DA-producing neurons from apoptosis. These observations indicate that the synthesized eco-friendly IONPs could be considered a promising adjuvant therapy against PD. The preclinical results suggest that environmentally friendly IONPs merit further translational evaluation as complementary treatments for PD, potentially reducing both functional dependence and nursing care demand if supported by clinical trials.

## Data Availability

The datasets presented in this study can be found in online repositories. The names of the repository/repositories and accession number(s) can be found in the article/supplementary material.
